# Significance of MDR1 and multiple drug resistance in refractory human epileptic brain

**DOI:** 10.1186/1741-7015-2-37

**Published:** 2004-10-09

**Authors:** Nicola Marchi, Kerri L Hallene, Kelly M Kight, Luca Cucullo, Gabriel Moddel, William Bingaman, Gabriele Dini, Annamaria Vezzani, Damir Janigro

**Affiliations:** 1 Cerebrovascular Research Center, The Cleveland Clinic, Cleveland, OH, 44195, USA; 2 Department of Neurological Surgery, The Cleveland Clinic, Cleveland, OH, 44195, USA; 3 Department of Neuroscience, Mario Negri Institute for Pharmacological Research, Milano, Italy

## Abstract

**Background:**

The multiple drug resistance protein (MDR1/P-glycoprotein) is overexpressed in glia and blood-brain barrier (BBB) endothelium in drug refractory human epileptic tissue. Since various antiepileptic drugs (AEDs) can act as substrates for MDR1, the enhanced expression/function of this protein may increase their active extrusion from the brain, resulting in decreased responsiveness to AEDs.

**Methods:**

Human drug resistant epileptic brain tissues were collected after surgical resection. Astrocyte cell cultures were established from these tissues, and commercially available normal human astrocytes were used as controls. Uptake of fluorescent doxorubicin and radioactive-labeled Phenytoin was measured in the two cell populations, and the effect of MDR1 blockers was evaluated.

Frozen human epileptic brain tissue slices were double immunostained to locate MDR1 in neurons and glia. Other slices were exposed to toxic concentrations of Phenytoin to study cell viability in the presence or absence of a specific MDR1 blocker.

**Results:**

MDR1 was overexpressed in blood vessels, astrocytes and neurons in human epileptic drug-resistant brain. In addition, MDR1-mediated cellular drug extrusion was increased in human 'epileptic' astrocytes compared to 'normal' ones. Concomitantly, cell viability in the presence of cytotoxic compounds was increased.

**Conclusions:**

Overexpression of MDR1 in different cell types in drug-resistant epileptic human brain leads to functional alterations, not all of which are linked to drug pharmacokinetics. In particular, the modulation of glioneuronal MDR1 function in epileptic brain in the presence of toxic concentrations of xenobiotics may constitute a novel cytoprotective mechanism.

## Background

Failure to respond to therapeutic concentrations of antiepileptic drugs (AEDs) is the usual basis for defining multiple drug resistant epilepsy, but the mechanisms underlying resistance to AEDs are still largely unknown. It is generally believed to be a multifactorial phenomenon, depending on both pharmacodynamic and pharmacokinetic mechanisms. The electrical and synaptic properties of neurons in epileptic human tissue are reported to undergo changes that may result in decreased susceptibility to some AEDs [1]. Multidrug resistance in epilepsy may also result from inadequate intraparenchymal AED concentrations due to poor penetration of the blood-brain barrier (BBB).

Recent findings suggest that the molecular mechanisms of clinically defined multiple drug resistance involve drug-efflux transporters such as the ATP-binding cassette subfamily B member 1 (ABCB1), also known as MDR1 or P-glycoprotein (P-gp) [2-4]. Specifically, in epileptic brain, genes associated with multiple drug resistance are overexpressed in the endothelial cells that constitute the BBB [5,6]. MDR1 is also overexpressed in neurons [4,7] and astrocytes (in which MDR1 is normally not measurable) from lesions associated with active epileptogenic foci in human and rodent brain [8-10].

The link between MDR1 overexpression and drug resistance in epilepsy is still poorly understood [6,11]. Thus, while localization of the drug extrusion pump in the BBB is consistent with reduced penetration of AEDs into the CNS, it is not known if or how the presence of MDR1 in the parenchyma affects drug delivery and distribution, or whether it is involved in different cellular functions. Evidence from several groups suggests that MDR1 diminishes the apoptotic response induced by growth factor withdrawal [12], decreases complement-mediated cytotoxicity ([13] and impairs the activation of caspase-dependent cell death pathways [14,15]. Reports indicate that all these events occur in epileptic tissue, and they are thought to be at least partly responsible for seizure-associated neuronal cell death. In addition, we recently found that epileptic astrocytes that overexpress MDR1 are devoid of p53, a proapoptotic factor [8].

In this study we have evaluated the relationship between neuronal and astrocytic MDR1 expression and the capacity of the cells to survive cytotoxic insults, using drug resistant epileptic human specimens. Our results suggest that overexpression of MDR1 leads to functional alterations in the CNS that may be linked to both drug pharmacokinetics and neuroglial survival in injured brain.

## Methods

### Human tissue

Human subjects were used as donors of cortical tissue samples. The investigation conformed to the principles outlined in the Declaration of Helsinki. Patient consent was obtained as per Institutional Review Board instructions before collection of the specimens. All the experiments involved small portions of human neocortical or hippocampal tissue, which were excised for therapeutic reasons from patients with pharmacoresistant epilepsy (see Table 1 for patient identification). Temporal lobe tissue was taken from the inferior or middle temporal gyrus during standard temporal lobectomy; frontal or parietal lobe samples were chosen from the most epileptogenic areas, as determined by chronic subdural grid or intraoperative electrocorticographic (EEG) recordings. Handling of the excised tissue depended on the experiment to be conducted, as described below.

### Cell isolation and primary cultures

Astrocyte cell cultures were established, as described by Marroni et al. [8,9,16], from cerebral cortical tissue obtained during temporal lobectomies (n = 3) conducted to relieve medically intractable seizures (see Table 1 for details on patients, ID# 12, 13, 14). The cells were passaged up to three times before use. MDR1 expression was not affected by the number of passages [8,9,16].

Commercially available normal human astrocytes were used as controls (ACBRI 371, Applied Cell Biology Research Institute, Kirkland, WA, USA, and Clonetics, Biowhitaker, Walkersville, MD, USA).

### Immunohistochemistry

To investigate MDR1 protein expression and cellular distribution in human epileptic tissue, slide-mounted sections (10 μm thickness) from frozen brain tissue (Table 1, patient ID# 1–11; 3 slices per patient) were double immunostained as previously described [8,9,16]. The primary antibodies used were mouse monoclonal anti-P-Glycoprotein (C494) (1:40, Calbiochem-Novabiochem Corporation, San Diego, CA, USA), human polyclonal anti-P-Glycoprotein (1:100, Calbiochem-Novabiochem Corporation, San Diego, CA, USA), mouse monoclonal anti-neuronal nuclei (NeuN) (1:500, Chemicon International, Temecula, CA, USA), rabbit polyclonal anti-neurofilament (NF) (1:200, Chemicon International, Temecula, CA, USA) and rabbit anti-cow glial fibrillary acidic protein (GFAP) (1:200, DAKO Corporation, Carpinteria, CA, USA). Secondary antibodies were chosen according to the primary antibody hosts: Texas red dye-conjugated affinipure donkey anti-mouse IgG (1:50, Jackson Immunoresearch Laboratories Inc., West Grove, PA, USA), and Fluorescein isothiocyanate (FITC)-conjugated affinipure donkey anti-mouse and anti-rabbit IgG (1:200, Jackson Immunoresearch Laboratories Inc., West Grove, PA, USA). Sections were coverslipped on glass slides using Vectashield mounting medium with DAPI (Vector, Burlingame, CA, USA) and analyzed by fluorescent microscopy.

### Cell counting

**Figure 1:**
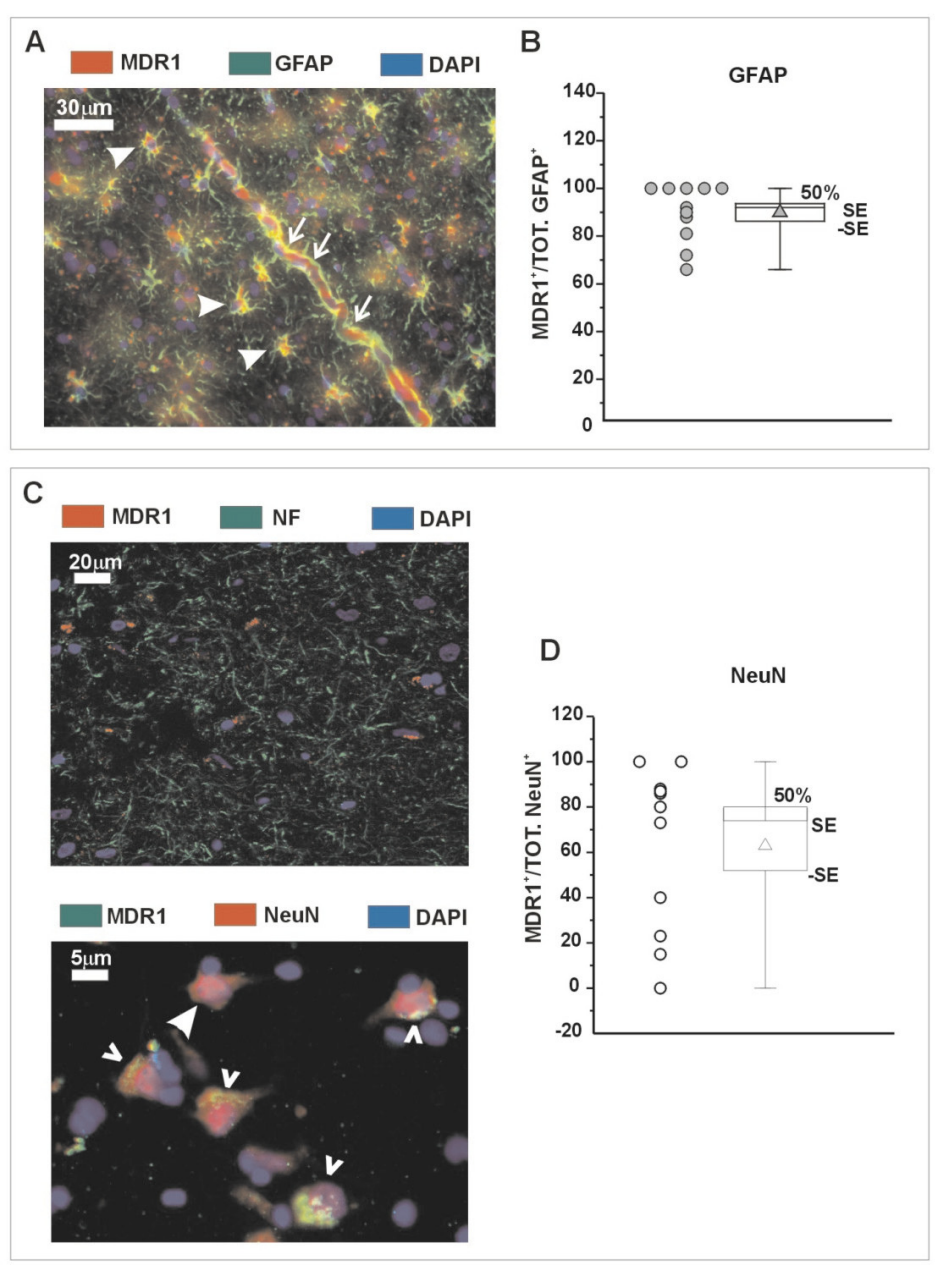
**Immunohistochemical detection of MDR1 expression in human drug-refractory epileptic brain. **Panels A-B show MDR1 expression at the BBB. Widespread GFAP immunoreactivity (*green*) co-localizes with MDR1 (*red*). Nuclei are stained *blue *with DAPI. Note that both parenchymal and perivascular astrocytes express MDR1 (arrowheads), as do endothelial cells of the brain capillaries (*thin arrows*). The box graph in B illustrates the percentage of GFAP-positive astrocytes (*GFAP*) in samples from 11 patients (Table 1, 1–11) that also expressed MDR1. Data points (*circles*), mean value (*triangle*), range (*horizontal bars*) and standard errors (° SE) are shown together with the 50^th ^percentile value. Panels C-D show the neuronal expression of MDR1. Double immunostaining with MDR1 and two neuronal markers (neurofilament, *NF *and NeuN) reveals that MDR1 is expressed in a subpopulation of epileptic neurons. *Arrows *point to MDR1 negative neurons, while *arrowheads *indicate the more frequently-occurring MDR1 positive neurons. Approximately 64% of cortical neurons (n = 264) were positive for MDR1. Quantitative analysis in D was obtained from 11 patients. Patient values (*circles*), mean (*triangle*), range (*horizontal bars*) and standard errors (± SE) are shown. The 50^th ^percentile is also indicated in the box graph.

For quantitative evaluation of neurons and astrocytes expressing MDR1 in epileptic tissue, we chose three 1600 μm^2 ^fields at random in each tissue slice (3 slices per patient, n = 11 patients; see Table 1, patient ID# 1–11). Within each field, NeuN or GFAP positive cells co-expressing MDR1 were counted using NIH Software and Photoshop 6 (Adobe), and the number was expressed as a fraction of the total number of neurons and astrocytes, respectively, in that field. Numbers for each field were then averaged for every slice, and the mean value for each patient is given in Fig. 1B,1D.

### Doxorubicin uptake

Astrocytes from epileptic tissue (Table 1, patient ID# 12, 13, 14) or commercially available control human astrocytes were cultured in 8-well chamber-slides. At confluence, the cells (approximately 8 × 10^4 ^per well) were treated overnight with 1μM XR9576, a specific MDR1 blocker [17,18], or for 2 h with 50 μM verapamil, a non-specific MDR1 blocker [19,20]. Further sets of astrocytes from epileptic tissue and controls were incubated as above but in the absence of blockers. Fluorescent red doxorubicin [21] was then added to all the cultures for 1, 2, 3, 5, 7 and 24 h. Incubation was stopped by rinsing twice with PBS and the cells were fixed overnight at 4°C in 4% formalin in PBS (pH 7.4). They were mounted and coverslipped the next day using Vectashield mounting medium. Doxorubicin uptake was analyzed by fluorescent microscopy (Leica Leitz DM-RXE, Wetzlar, Germany) using NIH Image Software. For each well, four fields (1600 μm^2^) were randomly analyzed and optical density measurements were averaged to obtain representative data. To quantify the doxorubicin in the cells, the fluorescence in each sample was compared with standard solutions of doxorubicin (10 nM-10 μM). Data were analyzed using Origin Lab 7 software.

### ^14^C-Phenytoin uptake

^14^C-Phenytoin uptake was measured in cultured astrocytes from epileptic tissue (Table 1, patient ID# 12, 13, 14) and in control astrocytes, as described by Meyer et al. [22]. The cells were cultured in 30-mm Petri dishes (approximately 10^6 ^cells) and incubated at 37°C with 1 ml of T3 cell buffer (Tris-HCl 50 mM, NaCl 120 mM, KCl 50 mM, pH 7.4) containing 10 μM ^14^C-Phenytoin (Specific activity = 9.57 μCi/μmol). The incubation was terminated after 10 s, 30 s, 1 min, 5 min or 10 min by adding 1 ml of ice-cold T3 buffer. The cells were washed with 1 ml of ice-cold T3 buffer and solubilized with 1% (w/v) Triton X-100 for 1 h at 37°C. Intracellular radioactivity was measured using a scintillation cocktail (Packard Ultima Gold, ECN, Costa Mesa, CA, USA).

### Acute in vitro toxicity

Brain slices (500 μm) were cut using a vibratome from dysplastic human cortex and temporal lobe epilepsy brain specimens (Table 1, patient ID# 9,10,11). Slices (n = 6 per patient) were maintained *in vitro *as previously described [23,24]. A further set of 6 slices was obtained from 3 naïve rats as previously described [25]. Slices (n = 2 per patient per experimental condition) were incubated for 2 h in artificial CSF containing, in mM: NaCl 124; KCl 3; CaCl_2 _1; MgCl_2 _1.4; NaHCO_3_^- ^26; KH_2_PO_4 _1.25; glucose 10, with or without 3 μM XR9576. A toxic concentration (375 μM) of Phenytoin (PHE) was then added for 5 h [22]. An additional set of slices was kept for 7 h in artificial CSF only, and electrophysiological measurement of activity was used to assess tissue viability throughout the experiment. Live/Dead Viability/Cytotoxicity solution (Molecular Probes L3224, Eugene, OR, USA) was used to quantify cell death or survival; EthD-1, a component of the solution, enters cell nuclei through damaged membranes, producing a bright red fluorescence in dead cells. The 2 mM EthD-1 stock solution (20 μl) was added to 10 ml of 1X sterile PBS. At the end of the experiment, each slice was washed with PBS and incubated with 2 ml of the above solution for 10 min under continuous oxygenation. The slices were then fixed in 4% formalin in PBS overnight at 4°C and cryoprotected by 30% sucrose. Three 20 μm slices were cryosectioned from each 500 μm slice and analyzed by fluorescent microscopy. DAPI was used to assess the total number of cells per slice, and GFAP and NeuN were used to identify glia and neurons (see 'Immunohistochemistry'). The number of EthD-1 positive cells was reckoned in 3 randomly chosen fields (1600 μm^2^) within each slice. These values were averaged for each slice and expressed as percentages of the average number of DAPI-positive cells in the chosen fields.

### Evaluation of glioneuronal damage

To evaluate the relationship between MDR1 positive cells and nuclear morphology (DAPI staining), 10 μm slices from 11 patients (Table 1, patient ID# 1–11) were obtained as described in 'Immunocytochemistry'. Small and condensed nuclei indicate apoptosis or irreversible cell damage, while large nuclei with diffuse DNA (DAPI) staining are typical of healthy cells. MDR1 positive cells with diffuse DNA staining (healthy cells) were counted over three randomly chosen fields in each slice and analyzed using NIH Software and Photoshop. Numbers for each field were averaged for every slice; the corresponding mean value is given in figure 3B.

### Statistical analysis of data

Data were expressed as means ± SEM. The level of significance between means was estimated by ANOVA (Origin 6.0. Microcal). Differences with p < 0.05 were considered significant.

## Results

Results were obtained from 14 patients (50% female) affected by intractable seizures. The mean patient age was 16.4 years (range 8 months – 49 years; SE = 4.6). For details, see Table 1.

### MDR1 expression in human epileptic brain tissue

Figure 1 shows representative micrographs of MDR1 expression in endothelial, glial and neuronal cells from epileptic human brain specimens (Table 1, patient ID# 1–11). The findings are consistent with previous work demonstrating that MDR1 expression in multiple drug resistant brains is confined to the cortical lesion site [4,7-9,16]; this was consistently observed in all epileptic samples in the present study. Thus, relatively normal brain distant from the dysplastic tissue can be used as "control". Abundant MDR1 immunopositive endothelial cells (*thin arrows in A*) were observed in epileptic cortex, as well as immunopositive parenchymal and perivascular astrocytes (*arrowheads in A*) double-labeled with GFAP, a specific glial marker. Out of 107 GFAP-positive astrocytes, 91 (85%) showed MDR1 staining (Figure 1B). We also examined MDR1 expression in neurons from epileptic tissue, identified by NeuN and NF immunoreactivity (Fig. 1C). Approximately 169 out of 264 NeuN-positive neurons (64%) showed MDR1 staining (Figure 1D).

### Role of MDR1 expression in drug extrusion by astrocytes

**Figure 2:**
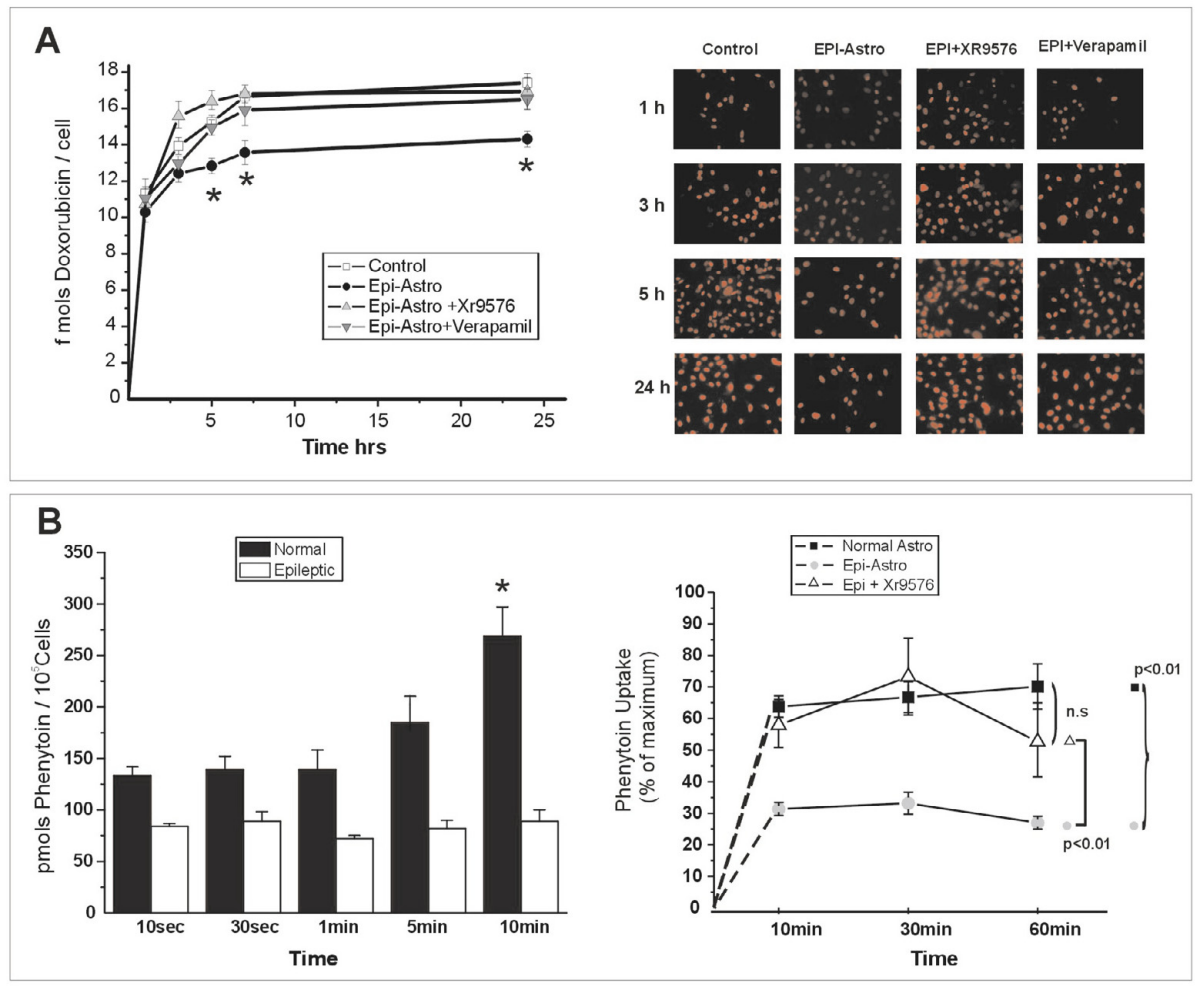
**Uptake of doxorubicin and phenytoin by astrocytes: effect of MDR1 blockade. **Panel A shows 1 μM doxorubicin uptake in astrocytes from epileptic human brain (n = 3 patients) and controls. Note that uptake was decreased in "epileptic" astrocytes compared to normal astrocytes, and this difference was abolished when MDR1 inhibitors (1 μM XR9576 or 50 μM verapamil) were added. Micrographs depict red fluorescent doxorubicin uptake in the various experimental conditions as assessed by fluorescence microscopy. Panel B shows the time-course of intracellular accumulation of ^14^C-Phenytoin in "epileptic" astrocytes and controls. Note that ^14^C-Phenytoin uptake into epileptic astrocytes reached a plateau after 10 s incubation, while uptake in control astrocytes was greater at each time point and reached a plateau after 10 min incubation (p < 0.01 vs. control; n = 3). ^14^C-Phenytoin uptake in "epileptic" astrocytes returned to control level in the presence of 1 μM XR9576 (°p < 0.01 vs. control''; ^p < = 0.01 vs. "epileptic'' astrocytes+XR9576).

To determine whether MDR1 expression in 'epileptic' astrocytes results in enhanced extrusion of xenobiotics compared to 'normal' astrocytes, we measured the cellular uptake of PHE and doxorubicin, two established substrates of P-glycoprotein [19,20,26,27] (Table 1, patient ID# 12, 13, 14; Fig. 2). We used 1 μM red fluorescent doxorubicin [21] and visualized its cellular distribution by fluorescent microscopy (Fig. 2A). We previously showed by western blot analysis that 'normal' astrocytes have lower levels of P-gp than 'epileptic' astrocytes [8,9]. Doxorubicin uptake was reduced in astrocytes from epileptic tissue compared to control astrocytes (p < 0.05). This effect was abolished by the specific blocker XR9576 (1 μM) [17,18], or by the less specific antagonist verapamil (50 μM), indicating active MDR1-mediated extrusion of doxorubicin by epileptic glia.

Fig. 2B shows that uptake of 10 μM^ 14^C-Phenytoin was significantly lower in astrocytes from epileptic tissue than in control astrocytes, and the difference was maximal after 10 min incubation with the AED (p < 0.05). The difference was abolished in the presence of 1 μM XR9576, indicating MDR1-mediated efflux of PHE in epileptic astrocytes. The MDR1 blockers did not affect doxorubicin or Phenytoin uptake in control astrocytes (not shown). Neither Phenytoin nor doxorubicin treatment induced toxicity in cell cultures under our experimental conditions.

### MDR1 and cell survival

**Figure 3:**
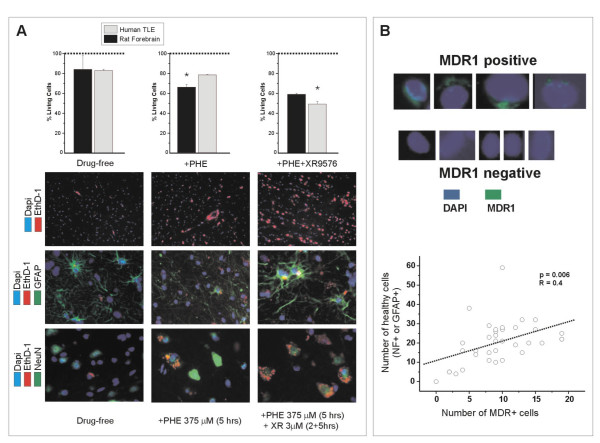
**Glioneuronal MDR1 expression and resistance to Phenytoin induced cytotoxicity. **Histograms in A show data (mean ± SE, n = 3) obtained using cortical slices from human epileptics (Table 1, ID# 9,10,11), or naïve rat cortical slices, treated for 5 h with 375 μM Phenytoin (PHE) ± 2 h preincubation with 3 μM XR9576. Note that PHE toxicity (as illustrated in the micrographs) was apparent in normal rat brain slices, and was greatly exacerbated in human epileptic tissue treated with the MDR1 blocker (p < 0.01). Micrographs depict immunohistochemical evidence of PHE-induced cytotoxicity in GFAP-positive astrocytes and NeuN-positive neurons as assessed by their co-localization with EthD-1, a marker of cell damage. All the cells (astrocytes and neurons) were assessed by DAPI staining. Note that the combination of PHE + XR9576 increased the percentage of injured cells (*red*) as compared to PHE alone. Panel B shows enlarged nuclei (identified by DAPI) in cells expressing (MDR1 positive) or not expressing (MDR1 negative) MDR1 protein. Note that small condensed nuclei (seen in MDR1 negative cells) reflect apoptosis or irreversible cell damage. In contrast, large nuclei with diffuse DNA staining (seen in MDR1 positive cells) are typical of healthy cells. The graph shows the positive correlation between cells with healthy nuclei and MDR1 expression (n = 34 independent values from slices obtained from 11 patients; p < 0.006).

MDR1 is involved in detoxification mechanisms that protect cells from xenobiotics [28], apoptosis [12] and other cellular stresses [13-15]. To investigate whether MDR1 expression in astrocytes and neurons from epileptic tissue enhances survival of toxic or injurious events, we exposed neocortical slices from human epileptic (Table 1, patient ID# 9, 10, 11) and rat brain to concentrations of PHE higher than those reported to induce toxicity in cultured rat astrocytes [22]. Bar histograms (Figure 3A) indicate that slices of human epileptic cortex exposed for 5 h to 375 μM PHE showed no cellular toxicity, as assessed by co-localization of the nuclear marker DAPI with EthD-1, a marker of cell damage. Conversely, significant cell loss (p < 0.05) was observed in rat brain slices under these experimental conditions. When MDR1 activity was blocked by 3 μM XR9576, PHE induced cell damage in the human epileptic cortex to an extent similar to that in rat brain tissue. Neurons and astrocytes, identified using NeuN and GFAP respectively, were similarly affected by PHE (Fig. 3A). The MDR1 inhibitor XR9576 alone (3μM) did not affect tissue viability (data not shown).

Figure 3B shows a significant positive correlation (R = 0.4, p < 0.006) between MDR1 expression and cell integrity, as measured by morphological evaluation of nuclear condensation (DAPI staining) [29,30]. Quantification of the cells retaining nuclear DNA integrity showed that no DNA damage occurred in 85% of glia and 66% of neurons expressing MDR1, suggesting a novel role for MDR1 in protecting neurons and glia from toxic agents.

## Discussion

The main finding was that MDR1 might have different roles depending on the location in the brain where it is expressed. Thus, in addition to overexpression in endothelial cells, which is likely to affect the penetration of AEDs into the brain, MDR1 is also overexpressed in the parenchyma, where it might have a cytoprotective role, extruding otherwise toxic concentrations of xenobiotics from the intracellular compartment.

### MDR1 expression in epileptic brain

In agreement with previous findings, we report here that MDR1 is highly expressed in vessels of the BBB and in parenchymal cells (histochemically identified as astrocytes and neurons) in drug refractory epilepsy of different etiologies [4,7-9,11,27]. Pharmacological evidence suggests that MDR1 overexpression in blood vessels of the BBB has the crucial role of extruding AEDs from brain to blood [19,20,27,31], and this phenomenon may contribute to failure of antiepiletic treatments. In this study, we focused on the effects of MDR1 expression in parenchymal neurons and astrocytes from pharmacoresistant epileptic tissue.

### MDR1 expression in astrocytes: drug uptake studies

**Figure 4:**
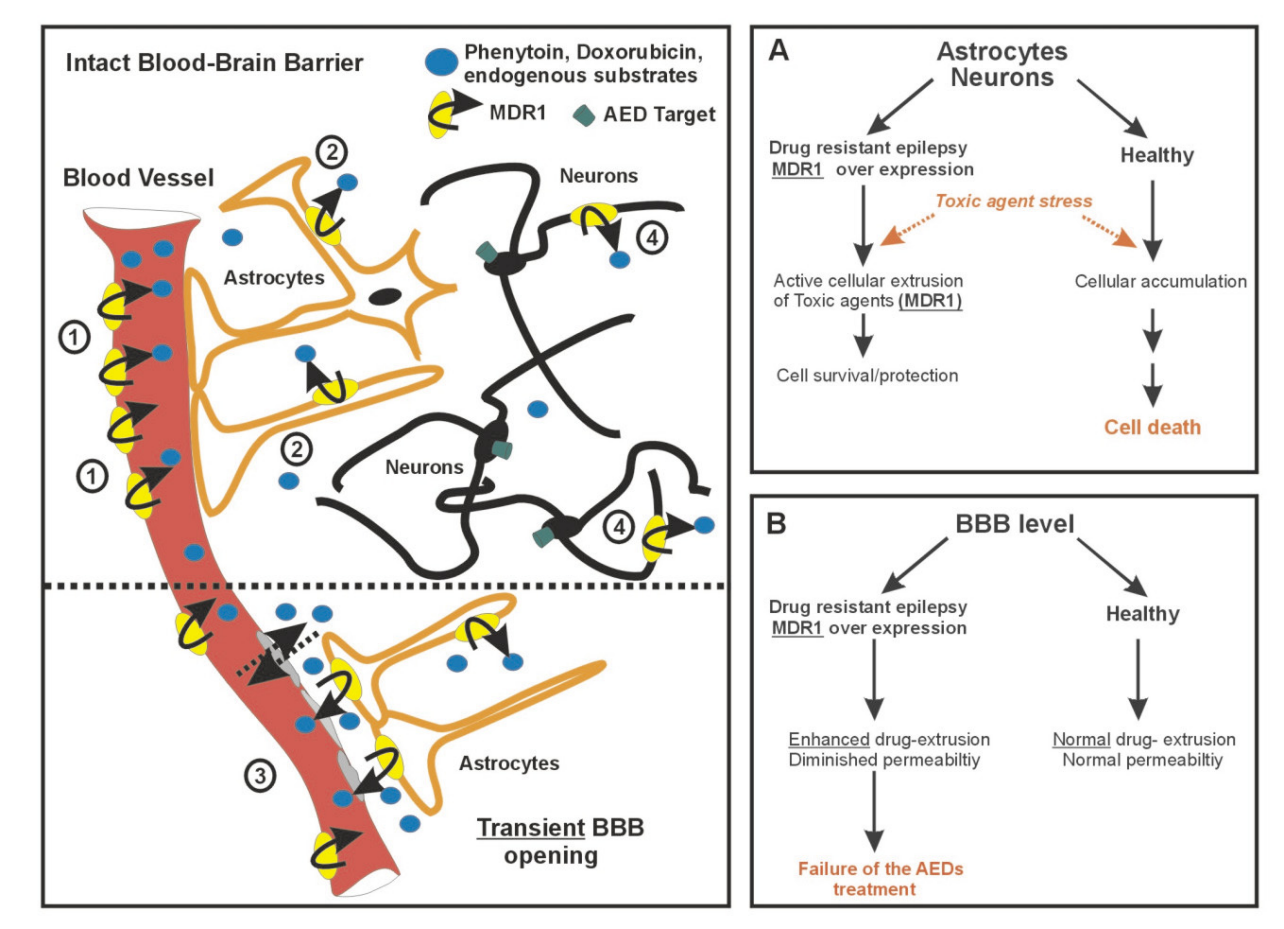
**Model of the proposed role of cell specific MDR1 expression in epileptic brain. **Under physiological conditions, when the blood-brain barrier is intact, overexpression of MDR1 (and possibly other drug resistance proteins [5,9]) in endothelial cells causes active net extrusion of drugs from the brain into the vascular lumen (left panel, (1)). A fraction of the AED molecules bypassing the MDR1 barrier (see Fig. 1A) will diffuse into the lipophilic membranes of parenchymal neurons and glia. MDR1 expression in these cells will lead to diminished intracellular sequestration of drugs and may increase their interstitial levels [27]. However, since the total amount of AED in the epileptic tissue is reduced in the first instance by active BBB extrusion, free AED in the extracellular space may still remain below therapeutic concentrations (left panel, (2) and (4)). In addition, MDR1 overexpression in parenchymal astrocytes and neurons affords protection against toxic concentrations of xenobiotics (left panel (2) and (4)). During the transient opening of the BBB due to epileptic activity, AEDs may be back-fluxed into the blood stream by MDR1 expressed at the glial end-feet of perivascular astrocytes, constituting a "second defense barrier" in the brain (left panel, (3)). Right panels A and B schematically summarize these possible roles of MDR1 in epileptic human brain.

We found that PHE and doxorubicin uptake by astrocytes from human epileptic tissue is reduced in comparison with normal astrocytes. The difference is abolished when MDR1 function is blocked, indicating that these drugs are efficiently extruded by "epileptic" astrocytes by a P-gp mediated mechanism. We suggest that this might contribute to decreasing the AED brain levels only if it occurs in perivascular astrocytes. Thus, *perivascular *astrocytes impinging on blood vessels may act as an additional barrier to drug penetration into the brain in regions where BBB permeability is transiently altered, for example during epileptic activity [32,33] (Fig. 4). In contrast, MDR1 overexpression by parenchymal glia would result in enhanced extrusion of substrates into the brain extracellular space; this is not compatible with a decrease in the concentration of AEDs at their neuronal targets, suggesting that it subserves a different function (see below).

### MDR1 expression in neurons and parenchymal glia: relationship to cell survival

We show in this paper that MDR1 is expressed in immunocytochemically identifiable neurons and astrocytes in brain slices from refractory human epilepsy patients. We have previously reported that basic apoptotic mechanisms may be defective in glia from epileptic tissue, since the pro-apoptotic proteins p53 and p21 could not be detected in "epileptic" astrocytes [8,9,16]. This evidence, together with overexpression of MDR1, suggests that "epileptic" astrocytes have gained a distinct survival advantage. This is supported by the marked enhancement of Phenytoin cytotoxicity in both glia and neurons when MDR1 function is blocked. The conclusion is consistent with the findings of Bittigau et al. [34] that various AEDs induce apoptotic neuronal cell death in developing naïve rat brain at plasma concentrations relevant to seizure control in humans; activators of MDR1 transcription prevented these effects.

Finally, we found a positive correlation between neuronal and astrocytic expression of MDR1 and lack of nuclear condensation, a marker of apoptosis and irreversible cell damage. Thus, expression of MDR1 in glia and neurons may protect the cells against toxic xenobiotics or against endogenous compounds that enter the brain in pathological conditions.

## Conclusions

In conclusion, as summarized in figure 4, our findings indicate a possible new function for MDR1. In normal brain, MDR1 operates at the blood-brain barrier, regulating the plasma/brain exchange of xenobiotics. In epileptic brain, the levels of astrocytic, neuronal and endothelial MDR1 are abnormal, possibly leading to altered brain penetration/distribution of drugs. Perivascular astrocytes may also contribute to this phenomenon. In addition to its drug extrusion effect at the BBB, which may be relevant for pharmacoresistance in epilepsy, this protein may have a role in neuroglial survival under hostile conditions such as those occurring in epileptic brain. The overexpression of multiple drug resistance could be the consequence of an altered mechanism of apoptotic cell death; this hypothesis is supported by the finding that changes in multiple drug resistance gene expression correlate with negative regulation of p53 and other pro-apoptotic genes [8].

## Abbreviations

AEDs: anti-epileptic drugs

BBB: blood-brain barrier

DOX: doxorubicin

GFAP: glial fibrillary acidic protein

MDR1: multiple drug resistance protein

NeuN: neuronal nuclei

NF: neurofilament

PHE: Phenytoin

## Competing interests

The authors declare that they have no competing interests.

## Author's contributions

NM and LC carried out the pharmacological experiments and data analysis.

KLH and KMK obtained surgical resections, established astrocytic cell cultures and performed the immunohistochemical and cell counting studies.

GM performed the acute in vitro toxicity experiments.

WB provided the surgical specimens.

GD provided additional input and editing.

AV and DJ designed and co-ordinated some of the experiments.

They also contributed equally to the preparation of the manuscript.

DJ, the PI of the study, co-ordinated and supervised most of the personnel involved in this project.

## Pre-publication history

The pre-publication history for this paper can be accessed here:



## Figures and Tables

**Table 1:** Information on patients affected by various forms of epilepsy whose brain specimens were used for the experiments (ICC = Immunocytochemistry and Immunohistochemistry, TOX = Phenytoin toxicity, UP = drug uptake).

**ID #**	**Sex**	**Age (years)**	**Use**	**Pathology**
1	M	8 months	ICC	Parieto-Occipital-Frontal cortical dysplasia, Right Hemispherectomy
2	F	49	ICC	Temporal Lobe Epilepsy, Hippocampal atrophy
3	F	10	ICC	Left Hippocampal sclerosis
4	F	3	ICC	Cortical Dysplasia, Right Hemispherectomy
5	M	7	ICC	Left Cortical Dysplasia
6	F	4	ICC	Right Frontoparietal Epilepsy
7	M	1	ICC	Left Frontal Lobe Epilepsy
8	M	25	ICC	Right Temporal Lobe Epilepsy
9	M	45	TOX, ICC	Temporal Lobe Epilepsy
10	F	4	TOX, ICC	Cortical Dysplasia
11	F	36	TOX, ICC	Temporal Lobe Epilepsy
12	M	24	UP	Left Frontal Lobe Epilepsy
13	F	14	UP	Cortical Dysplasia
14	M	7	UP	Left Temporal/Frontoparietal Epilepsy
